# The Heterogeneous Effects of *Epichloë* and *Rhizophagus irregularis* on the Physiological and Rhizosphere Microbial Community of *Festuca rubra*

**DOI:** 10.3390/plants15030467

**Published:** 2026-02-02

**Authors:** Yanying Zhou, Zhengming Luo, Xuerong Wang, Tong Jia

**Affiliations:** 1 Department of Life Sciences, Xinzhou Normal University, Xinzhou 034000, China; 18135051862@163.com (Y.Z.); luozhengming2004@126.com (Z.L.); 2 Soil Health Laboratory of Shanxi Province, Shanxi Agricultural University, Taiyuan 030031, China; 3 Shanxi Key Laboratory for Ecological Restoration of Loess Plateau, Institute of Loess Plateau, Shanxi University, Taiyuan 030031, China; wanxueron@163.com

**Keywords:** arbuscular mycorrhizal fungi, endophytic fungi, nitrogen, soil enzyme activity, soil microbial community

## Abstract

In nature, a significant number of plant species form symbiotic associations with microorganisms, with arbuscular mycorrhizal fungi (AMF) and endophytic fungi being two prevalent groups of these partners. However, the ability to establish such symbioses with AMF and endophytic fungi is limited to a small fraction of native grass species. Nitrogen is a crucial nutrient for plant growth, yet it is often a limiting factor, underscoring the importance of understanding how plants acquire it. AMF enhance plant growth by improving nitrogen uptake efficiency, but the combined effects of endophytic fungi and AMF on plant physiology and ecology remain underexplored. To address this knowledge gap, in the present study, we conducted an indoor randomized block experiment to investigate the influence of endophytic fungi and AMF infection on the physiological and ecological attributes of *Festuca rubra* under various nitrogen regimes. The findings indicated that AMF inoculation significantly affected the total carbon content of *F. rubra* and the total sulfur concentration in its underground tissues across different nitrogen conditions. Additionally, dual colonization by AMF and endophytic fungi had a significant impact on the underground total nitrogen content of the plants. Furthermore, the complex interactions among AMF, endophytic fungi, and nitrogen availability emerged as critical determinants influencing underground total carbon content, transpiration rates, intercellular carbon dioxide concentrations, and the activity of soil extracellular enzymes in *F. rubra*. The activity of soil extracellular enzymes and pH significantly affected the structure and diversity of rhizosphere bacterial, fungal, and archaeal communities. AMF enhanced the richness of rhizosphere bacterial communities under low-nitrogen conditions, whereas endophytic fungi infection increased bacterial diversity. Soil extracellular enzyme activity and pH were closely related to the community structures and diversities of rhizosphere bacteria, fungi, and archaea. This study clarifies the effects of AMF and endophytic fungi infection on the physiological and ecological characteristics of *F. rubra*, significantly contributing to our understanding of the synergistic mechanisms governing the interactions among AMF, endophytic fungi, and their host plants.

## 1. Introduction

Within natural ecosystems, plants form diverse associations with microorganisms, which can range from mutualistic to antagonistic in nature [1]. Two prevalent groups of symbiotic microorganisms associated with plant hosts are arbuscular mycorrhizal fungi (AMF), which primarily colonize the underground root system, and endophytic fungi, which are widely distributed in aboveground tissues. AMF, which are ubiquitously distributed in terrestrial ecosystems, form symbiotic associations with most terrestrial plants. The extraradical hyphal networks of AMF mediate plant–soil interactions, playing indispensable roles in enhancing ecosystem productivity, sustaining biodiversity, and maintaining soil health [1,2]. AMF are primarily classified within the phylum Glomeromycota. They exert significant effects on plant growth, development, and structural composition [2,3]. Research shows that AMF significantly increase leaf nitrogen and phosphorus content, elevate levels of chlorophyll, chlorophyll a, and chlorophyll b, and enhance the presence of soluble sugars and soluble proteins [2,3]. Additionally, AMF can improve various enzyme activities, affecting both foliar and soil enzymes [3]. Furthermore, AMF increase the net photosynthetic rate (27.7%) and transpiration rate (31.1%) in leaves [2]. In natural grasses, endophytic fungi establish symbiotic relationships with AMF and host plants, significantly influencing the physiological and ecological traits of the host [3].

Endophytic fungi reside within healthy plant tissues at some stage of their life cycle or throughout their entire existence without causing visible disease symptoms or significant harm to the host. As microorganisms inhabiting plant tissues, they are a natural component of the plant microbiome. By living long term in the specialized environment of plants, they co-evolve with their hosts [4,5]. *Festuca rubra*, a perennial herbaceous grass of the Poaceae family, is native to and widely distributed across the Northern Hemisphere, including temperate regions of Europe, Asia, and North America [6,7]. As a common host for endophytic fungi, its natural infection rate often exceeds 70–90% [8,9], with prior microscopic examinations confirming endophytic infection in experimental seeds. Extensive international studies have focused on its symbiotic mechanisms with microbes: Clay first highlighted its obligate symbiosis with endophytes that enhance stress tolerance [10]. The plant *F. rubra* maintains a symbiotic relationship with the vertically transmitted endophytic fungus *Epichloë festucae* [11]. This endophyte exerts a significant influence on the plant’s nutritional balance [11]. Furthermore, *E. festucae* plays a crucial role in fungal–plant interactions [12]. Endophytic fungi promote plant growth and biomass accumulation [4,5]. They also facilitate water and nutrient absorption, improve stress tolerance and adaptability, and play a crucial role in disease resistance by defending against pathogen invasion [13]. Although both arbuscular mycorrhizal fungi (AMF) and endophytic fungi maintain close relationships with plants, considerable uncertainty remains regarding the interactions among endophytic fungi, AMF, and host plants [14].

Moreover, endophytic fungi and AMF often co-colonize various plant species. Through their symbiotic relationships with plants, AMF play a crucial role in regulating soil microbial community structure, which in turn influences microbial diversity and community composition. Both endophytic fungi and AMF establish symbiotic mycorrhizal systems via plant roots, leading to the release of substances such as soil enzymes. This process activates mineral nutrients immobilized in the soil, thereby increasing their availability [15] and improving soil quality [16,17]. Research indicates that endophytic fungi and AMF can enhance soil nutrient content, specifically nitrogen and phosphorus, by increasing the activity of acid phosphatase and urease in the rhizosphere [18]. Soil properties such as pH, total nitrogen content, and soil organic carbon content are key factors influencing the structure of rhizosphere microbial communities [19].

In the context of global climate change, nitrogen deposition has garnered increasing attention. A significant scientific issue is the impact of varying nitrogen levels on plant–microbe interactions. Numerous studies have demonstrated that AMF can promote plant growth under low-nitrogen conditions to a certain extent [2,3]. Furthermore, AMF exhibit synergistic interactions with plants under increased global nitrogen deposition. For example, nitrogen addition and elevated CO_2_ concentrations significantly enhance the productivity of AM-inoculated plants compared to those without AMF symbiosis [20]. Endophytic fungal infections can enhance plant growth and competitive ability [21,22], increase tolerance to abiotic stresses such as nutrient deficiencies, and improve the diversity of host rhizosphere microbial communities [23,24]. From this perspective, the interactions among AMF, endophytic fungi, and host plants remain considerably uncertain. Based on this, we propose the following scientific hypotheses: (1) the effects of endophytic fungi and AMF on the physiological and ecological traits of host plants vary under different nitrogen levels, and (2) there is a synergistic interaction between endophytic fungi and AMF in shaping the rhizosphere microbial communities of host plants. The aim is to identify key ecological factors influencing plant–microbe interactions and to provide a scientific basis for a deeper understanding of the mechanisms underlying these interactions.

## 2. Results

### 2.1. Effects of Endophytic Fungi and AMF Infection on Nutrient and Chlorophyll Content

**Figure 1:**
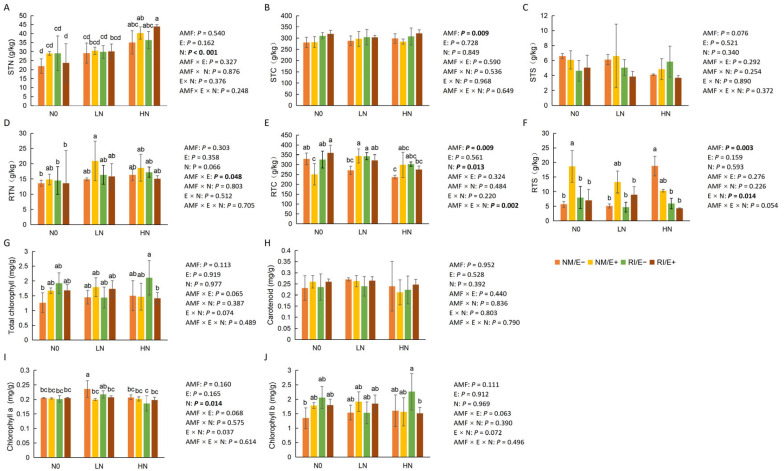
Effects of endophytic fungi and AMF on nutrient and chlorophyll contents of *F. rubra* under varying nitrogen levels (mean ± standard deviation). Note: Sample size (n = 3 per treatment), statistical tests (multi-way ANOVA followed by Duncan’s multiple-range test), and different lowercase letters indicate significant differences (*p* < 0.05), while values without labeled letters are not significantly different (*p* > 0.05). (**A**) STN, shoot total nitrogen; (**B**) STC, shoot total carbon; (**C**) STS, root total sulfur; (**D**) RTN, root total nitrogen; (**E**) RTC, root total carbon; (**F**) RTS, root total sulfur; (**G**) total chlorophyll content; (**H**) carotenoid content; (**I**) chlorophyll a content; (**J**) chlorophyll b content.

Multi-way ANOVA results indicated that the total nitrogen content in belowground plant tissues was influenced by dual infection with AMF and endophytic fungi (*p* < 0.05) (Figure 1D). Belowground total carbon content was significantly affected by AMF infection (Figure 1E). Moreover, under low-nitrogen conditions, both endophytic fungi and AMF significantly increased the RTC content in plants (Figure 1E). The total sulfur content in belowground tissues was influenced by the interaction between nitrogen levels and endophytic fungal infection. Specifically, endophytic fungal infection significantly enhanced the RTS content under the N_0_ treatment (Figure 1F). Regarding chlorophyll content, the results revealed that only nitrogen level exerted a significant effect on chlorophyll a content, with endophyte-free plants exhibiting significantly higher chlorophyll a content than endophyte-infected plants (Figure 1I).

### 2.2. Effects of Endophytic Fungi and AMF Infection on Photosynthetic Characteristics and Soil Enzyme Activity

**Figure 2:**
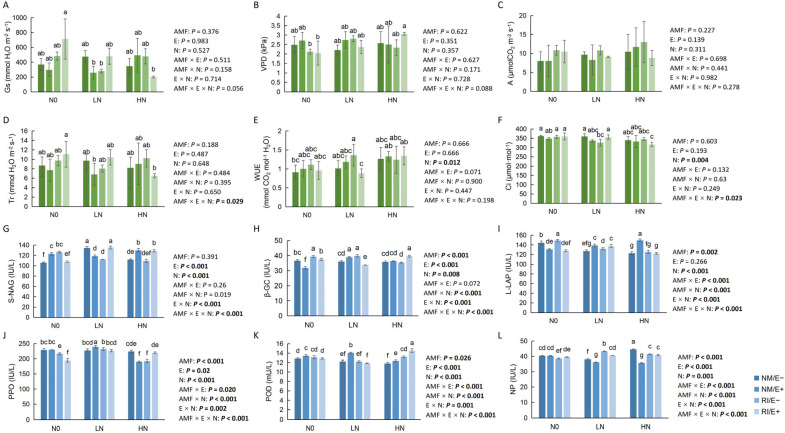
Effects of endophytic fungi and AMF on photosynthetic characteristics and enzyme activities in rhizosphere soil of *F. rubra* under different nitrogen levels (mean ± standard deviation). Note: Sample size (n = 3 per treatment), statistical tests (multi-way ANOVA followed by Duncan’s multiple-range test), and different lowercase letters indicate significant differences (*p* < 0.05), and the difference is not significant for those without labeled letters (*p* > 0.05). Green represents photosynthetic characteristics, and blue indicates enzymatic activity.(**A**) Gs, stomatal conductance; (**B**) VPD, vapor pressure deficit; (**C**) A, net photosynthetic rate; (**D**) Tr, transpiration rate; (**E**) WUE, water use efficiency; (**F**) Ci, intercellular CO_2_ concentration; (**G**) S -NAG, N-acetyl-β-D-glucosidase; (**H**) β-GC, β-glucosidase; (**I**) L-LAP, leucine aminopeptidase; (**J**) PPO, polyphenol oxidase; (**K**) POD, peroxidase; (**L**) NP, neutral phosphatase.

Different nitrogen levels had a significant impact on the carbon dioxide concentration (Ci) and water use efficiency (WUE) of *F*. *rubra* plants (*p* < 0.05). Simultaneously, the interaction among AMF infection, endophytic fungi, and varying nitrogen levels significantly influenced both the transpiration rate (Tr) and Ci of the plants (*p* < 0.05) (Figure 2). Leaf stomatal conductance (Gs) also exhibited significant variation across nitrogen treatments (*p* < 0.05). In the double-infection treatments, WUE was significantly lower in the low-nitrogen group compared to the high-nitrogen group (*p* < 0.05) (Figure 2).

Furthermore, the activity of soil extracellular enzymes was significantly influenced by AMF infection, endophytic fungi, different nitrogen levels, and their interactions. Specifically, AMF infection significantly affected soil *β*-glucosidase (*β*-GC, *p* < 0.001), polyphenol oxidase (PPO, *p* < 0.001), peroxidase (POD, *p* < 0.05), leucine aminopeptidase (L-LAP, *p* < 0.01), and neutral phosphatase (NP, *p* < 0.001). Endophytic fungi infection significantly affected N-acetyl-*β*-D-glucosidase (S-NAG, *p* < 0.001), *β*-GC (*p* < 0.001), PPO (*p* < 0.05), and NP (*p* < 0.001) in the rhizosphere soil. Concurrent infection with AMF and endophytic fungi significantly altered soil PPO (*p* < 0.05), POD (*p* < 0.001), L-LAP (*p* < 0.001), and NP (*p* < 0.001). In the no-nitrogen-addition treatment group, the AMF-infected group showed the highest soil *β*-glucosidase (β-GC) activity (*p* < 0.05) (Figure 2).

### 2.3. Composition and Diversity of Soil Microbial Communities in the Rhizosphere

**Figure 3:**
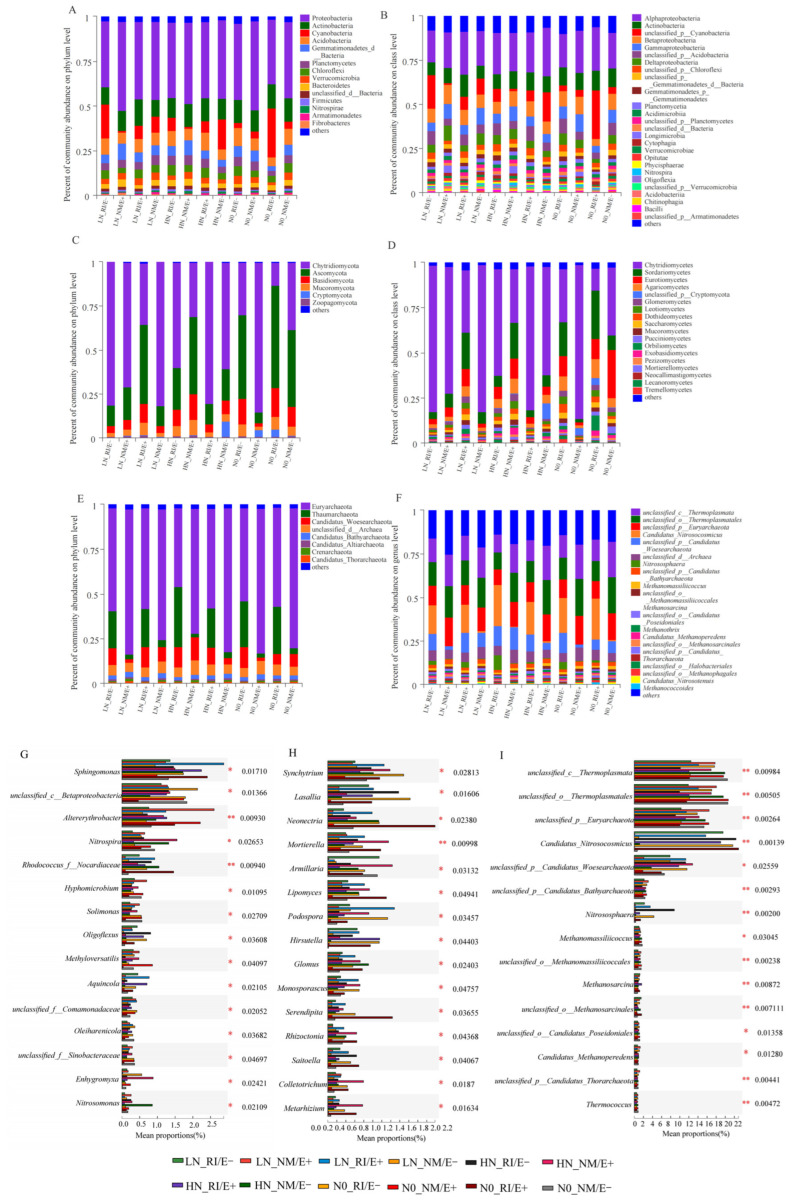
Analysis of the composition and differences in the microbial communities of F. rubra rhizosphere soil. Note: (**A**,**B**,**G**) Bacteria; (**C**,**D**,**H**) Fungi; (**I**,**E**,**F**) Archaea. Subfigures (**A**,**C**,**E**) display phylum level classification; Subfigures (**B**,**D**) display class level classification; Subfigure (**F**) displays genus level classification. “*” indicates a significance level of *p* < 0.05, “**” indicates a significance level of *p* < 0.01.

Based on the NCBI-NR database, the rhizosphere bacterial community of *F*. *rubra* comprised 153 phyla, 245 classes, 420 orders, 825 families, 3469 genera, and a total of 31,503 bacterial species. Among all bacterial groups, Proteobacteria exhibited the highest relative abundance, ranging from 40.5% to 48.2% across the four inoculation treatments (Figure 3A,B). In contrast, fungi were represented by 10 phyla, 42 classes, 107 orders, 283 families, 491 genera, and 976 species. Under varying inoculation treatments, Chytridiomycota exhibited the highest relative abundance (Figure 3C,D).

In the rhizosphere soil of *F*. *rubra*, archaea comprised 23 phyla, 41 classes, 67 orders, 105 families, 238 genera, and 1326 species, with the dominant phylum being Euryarchaeota, which reached a relative abundance of 76.9%. In treatments inoculated with AMF, Thaumarchaeota exhibited the highest relative abundance at 27.1% (Figure 3E,F). Significant differences (*p* < 0.05) were observed at the genus level among the bacterial, fungal, and archaeal communities in the rhizosphere soil across different nitrogen levels and inoculation treatments (Figure 3G–I).

**Figure 4:**
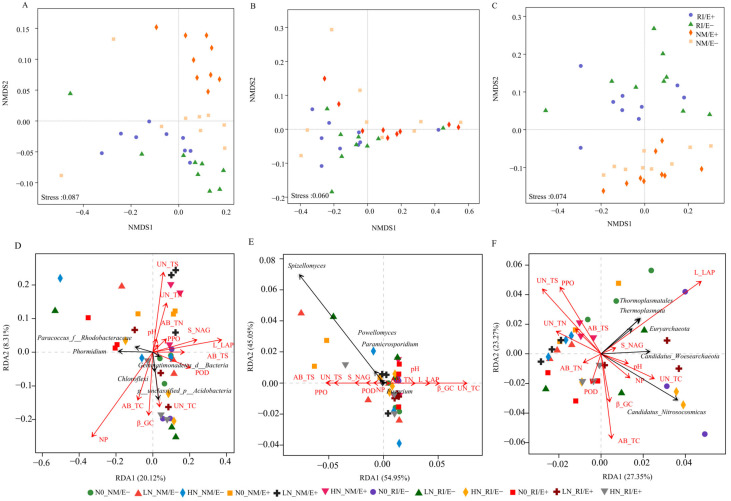
Community structures and redundancy analyses of the top 5 bacterial (**A**,**D**), fungal (**B**,**E**), and archaeal (**C**,**F**) communities at the genus level with environmental factors in the rhizosphere soil of *F. rubra*. Red arrows represent various ecological factors, while black arrows indicate dominant microorganisms.

AMF infection significantly affected the Shannon index of fungal and archaeal communities (*p* < 0.05). Additionally, nitrogen levels and AMF infection notably affected the Shannon and Simpson indices of fungal communities and the Sobs index of archaeal communities (*p* < 0.05). The interaction between nitrogen levels and AMF infection significantly influenced the Shannon indices of bacterial communities and the Sobs indices of archaeal communities (*p* < 0.05) (Table 1). The Sobs index of bacterial communities in rhizosphere soil peaked under the treatment with no nitrogen addition combined with AMF infection (*p* < 0.05). Furthermore, under low nitrogen levels with endophytic fungi infection, the Shannon index of rhizosphere bacterial communities was significantly higher than that of the control group (*p* < 0.05) (Table 2). These findings suggest that AMF promote bacterial community richness under low-nitrogen conditions, while endophytic fungi infection enhances bacterial diversity. Non-metric multidimensional scaling (NMDS) analysis revealed that, across different nitrogen levels, the infection by endophytic fungi and AMF led to significant differences in the rhizosphere microbial community structure of *F. rubra* (Figure 4).

### 2.4. Effects of Plant Nutrients and Soil Enzyme Activity on the Composition of Rhizosphere Microbial Communities

RDA analysis revealed that both soil extracellular enzyme activity and pH significantly influenced the rhizosphere microbial communities of *F. rubra*, with distinct key factors affecting bacteria, fungi, and archaea (Figure 4). Soil neutral phosphatase activity had the strongest impact on the dominant bacterial communities in the rhizosphere (Figure 4D). Additionally, soil β-glucocysteinyl-L-cysteine (β-GC) activity primarily affected the Acidobacteria community, while the genus *Phormidium* was primarily influenced by alkaline phosphatase activity (Figure 4D). In fungal communities, soil polyphenol oxidase activity had the most significant effect on the dominant fungal populations (Figure 4E). Lastly, soil L-LAP activity most significantly influenced the structure of the archaeal community (Figure 4F).

## 3. Discussion

Studies investigating the physiological and ecological characteristics of *F. rubra* under varying nitrogen levels and mycorrhizal infection treatments have demonstrated that infection with AMF significantly influences the total carbon and total underground sulfur content in the plants. Furthermore, research indicates that AMF can serve as a carbon reservoir within the plant rhizosphere [25], stimulate enhanced photosynthesis, and promote carbon accumulation in plants [26,27]. Additionally, AMF exert regulatory effects on plant nitrogen uptake, thereby reducing the net nitrogen acquisition cost for infected plants. AMF also affect soil nitrogen content; their absorption or release of nitrogen influences soil decomposers, ultimately impacting their carbon demand and, consequently, the total carbon content of plants. Moreover, related studies indicate that sulfur uptake in plants is regulated by fungi such as AMF [28]. AMF facilitate plants in efficiently absorbing more soil sulfur [29] and can cooperate with beneficial soil microorganisms, including sulfur-oxidizing bacteria, rhizobia, and phosphorus-solubilizing bacteria [30]. These beneficial microorganisms positively influence AMF growth and reproduction [31], which enhances mycorrhizal colonization rates. As a result, this promotes the uptake and transport of soil sulfur by AMF, improving sulfur nutrition for host plants and increasing the total underground sulfur content in plants [32].

In this study, simultaneous infection by AMF and endophytic fungi affected the total nitrogen content in plant roots. For example, this finding aligns with conclusions from related research on Phragmites australis [33]. AMF and endophytic fungi enhanced nitrogen uptake efficiency in low-nitrogen environments by co-regulating nitrogen transport protein activity and root morphogenesis [33]. This synergistic effect may stem from endophytic fungi secreting growth-regulating substances (e.g., indole-3-acetic acid) that promote root development, while the AMF mycelial network improves spatial nitrogen exploration in soil, thereby forming complementary nutrient capture strategies [34]. Meanwhile, varying nitrogen levels primarily influence above-ground total nitrogen and below-ground total carbon content. However, numerous inconsistencies remain regarding the mechanisms governing below-ground carbon deposition and transformation under nitrogen enrichment conditions. For instance, Curry et al. [35] studied plants in Scottish peatlands and found that nitrogen enrichment promoted carbon fixation. However, they observed that the underground allocation of photosynthetically assimilated carbon varied by plant functional type, which generally led to a reduction in the amount of carbon transferred to root exudates. In contrast, Ge et al. [36] reported that nitrogen application increased rice stem and root biomass, promoting the transfer of photosynthetically assimilated carbon to the rhizosphere and soil organic carbon. The interaction among three factors—infection by AMF, endophytic fungi, and varying nitrogen levels—significantly influences total underground carbon content in plants [37], which further mediates AMF’s effects on rhizosphere bacterial richness.

In this study, AMF enhance rhizosphere bacterial richness under low-nitrogen conditions but fail to exert this effect under high nitrogen conditions—a pattern rooted in context-dependent carbon allocation trade-offs and inter-microbial competition. Under LN, nitrogen limitation acts as a strong selective pressure that reinforces the mutualism between AMF and *F. rubra*. Plants prioritize carbon allocation to AMF as a functional strategy to expand nutrient foraging capacity, as AMF’s extraradical hyphal networks can access nitrogen pools unavailable to plant roots alone [38]. Herbaceous plants such as *F. rubra*, characterized by fibrous root systems, exhibit higher carbon allocation ratios due to their root structural traits [39]. AMF translocate these photosynthates (e.g., sugars, organic acids, and glomalin-related soil proteins) to the rhizosphere via hyphal networks, creating a carbon-rich microhabitat [40].

This carbon subsidy is pivotal for rhizosphere bacteria. The additional carbon input alleviates energy constraints for heterotrophic bacteria, facilitating the proliferation of diverse taxa such as Proteobacteria and Acidobacteria—groups closely associated with nutrient cycling. Moreover, AMF enhance the activity of soil enzymes like leucine aminopeptidase under LN [41], accelerating the mineralization of organic nitrogen into plant- and microbe-accessible forms. This creates a positive feedback loop: AMF-derived carbon supports bacterial growth, while bacteria contribute to nutrient mobilization, reinforcing the mutualistic tripartite interaction between *F. rubra*, AMF, and rhizosphere bacteria. AMF also modify soil physical properties by promoting aggregate stability, which increases niche diversity and supports higher bacterial richness [42].

In contrast, HN conditions disrupt this feedback loop through multiple interrelated mechanisms. HN alleviates nitrogen limitation, triggering a carbon-nitrogen trade-off where plants reduce carbon allocation to AMF [43]. This reduction in carbon investment suppresses AMF hyphal growth and exudation, diminishing the carbon subsidy to rhizosphere bacteria [43]. *Rhizophagus irregularis* (RI), in particular, exhibits a negative correlation with soil nitrogen availability, further reducing its hyphal biomass and carbon secretion under HN [43]. Moreover, HN intensifies inter-microbial competition for limited resources, and HN-induced shifts in soil pH and nutrient stoichiometry exacerbate this competition by altering the competitive advantage of AMF over bacteria.

The photosynthetic characteristics of *F. rubra* plants are significantly influenced by different nitrogen levels, which affect both intercellular CO_2_ concentration and water use efficiency. These findings are consistent with numerous previous studies. Additionally, research has shown that mature cassava leaves exhibit markedly different photosynthetic rates depending on nitrogen levels [44]. Results on soil enzyme activity in *F. rubra* indicate that the interaction among AMF infection, endophytic fungi, and varying nitrogen levels significantly influences plant transpiration rates, intercellular CO_2_ concentrations, and soil extracellular enzyme activity. Previous studies have established a positive correlation between nitrogen fertilization and plant photosynthetic efficiency. Nitrogen primarily regulates various physiological processes, including plant hormone metabolism, chlorophyll degradation, nucleic acid and protein degradation, nitrogen and lipid metabolism, antioxidant activity, aging-related enzymes, and transcription factors [45]. Research further indicates that as nitrogen application increases, the net photosynthetic rate, transpiration rate, and water use efficiency of barnyard grass initially rise to a peak before declining. In contrast, intercellular CO_2_ concentration generally increases, while stomatal conductance shows no significant change [46].

In previous studies, inoculation with AMF significantly increased the activity of alkaline phosphatase, sucrase, and catalase in plant rhizosphere soil [2,3]. This study also found that AMF is significantly related to soil peroxidase activity. The acidic phosphatase secreted by AMF was closely associated with enhanced soil phosphorus availability [2,3]. Changes in peroxidase activity may reflect AMF’s involvement in lignin degradation or reactive oxygen species scavenging, indirectly influencing soil carbon stability and the oxidative stress status of plants [47]. Consistent with these findings, numerous studies indicate that varying nitrogen levels substantially impact soil enzyme activity. Specifically, soil enzyme activity is closely correlated with nitrogen application rates, with a significant positive correlation observed between soil urease activity and alkaline-hydrolyzable nitrogen content [48]. Additionally, studies have revealed significant differences in soil urease, nitrate reductase, and nitrite reductase activities under different nitrogen fertilizer levels, with all three enzymes showing a clear upward trend as nitrogen application increases [49]. The application of endophytic fungi enhances sucrase and catalase activities in peanut continuous-cropping soils [50]. These findings provide valuable data that enhance our understanding of the biotic and abiotic factors influencing plant–microbe interactions and establish a scientific foundation for elucidating the mechanisms underlying these interactions.

Several factors influence the relationship between soil microbial communities and enzyme activity, including nitrogen fertilizer application, soil physicochemical properties, plant species, and cultivation management practices [51]. Soil enzyme activity is a key microbial factor involved in nutrient transformation and cycling [52]. This study identifies the primary enzyme activities that influence the dominant community structures of bacteria, fungi, and archaea in the rhizosphere soil of *F. rubra*. Specifically, soil neutral phosphatase, polyphenol oxidase, and leucine aminopeptidase are the key enzymes associated with bacteria, fungi, and archaea, respectively. Changes in these soil enzyme activities can impose microbial constraints on the availability of related nutrients [53]. Research indicates that soil enzyme activity is closely related to bacterial diversity [53]. The composition and diversity of soil microbial communities are also strongly linked to these enzyme activities, with multiple environmental factors regulating this relationship. Furthermore, physicochemical properties such as soil organic matter content, pH, and nutrient levels play a significant role in influencing both microbial communities and enzyme activities [54]. The impact of soil physicochemical properties on microbial communities varies across soil types [55,56]. Nitrogen addition affects microbial community structure and enzyme activity by reducing soil pH, which may alter microbial substrate utilization strategies and metabolic pathways [57]. In this study, nitrogen addition may have partially alleviated the negative correlation observed between actinomycete abundance and pH.

It is important to acknowledge certain limitations of this study that should be addressed in future research. First, this study employed indoor pot-controlled experiments, which differ significantly from natural field habitats. Factors such as the spatiotemporal heterogeneity of nitrogen deposition, the native diversity of soil microbial communities, interactions among plant communities, and climate fluctuations in field environments may influence the interaction patterns between plants and microorganisms. Therefore, the applicability of this study’s conclusions to field conditions requires further validation. Future research could enhance the generalizability of these findings by combining pot experiments with long-term field studies. Second, the analysis of microbial communities in this study primarily focused on species richness and changes in core taxa, without an in-depth investigation of microbial functional gene expression, metabolic pathway activity, or interaction networks of key functional microbial groups. This limits the comprehensiveness of the interpretation of the molecular mechanisms by which endophytic fungi and AMF regulate rhizosphere microbial communities. Subsequent studies should integrate multi-omics approaches, such as metatranscriptomics and metabolomics, to elucidate plant–microbe interaction mechanisms under nitrogen gradients from a functional perspective. This would provide more detailed theoretical support for understanding the responses of plant–microbial symbiotic systems to nitrogen deposition.

## 4. Materials and Methods

### 4.1. Research Materials and Experimental Design

**Figure 5:**
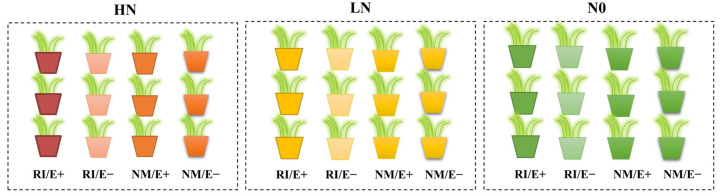
Schematic diagram of the experimental design. Note: RI: *R. irregularis* colonization; NM: no *R. irregularis* colonization; E+: *E. festucae* infected; E−: *E. festucae* free; Nitrogen treatment: HN:high-nitrogen; LN: low-nitrogen; N0: no nitrogen.

The host plant selected was *F. rubra*. Prior to the experiment, the infection rate of endophytic fungi in *F. rubra* seeds was assessed via microscopic examination. To prepare the seeds for planting, they were surface-sterilized by soaking in a 10% H_2_O_2_ solution for 10 min, followed by rinsing with sterile water. The seeds were then dried at 60 °C for one month using an oven-drying method to ensure they were free of endophytic fungal infection. Complete sterilization of the seeds was confirmed through aniline blue staining [6]. The plant growth medium consisted of river sand and zeolite, both sieved through a 2 mm mesh. These components were mixed in a specific ratio and sterilized in an autoclave at 121 °C and 0.11 MPa for 2 h to create a sterile environment and eliminate interference from other microorganisms. *R. irregularis* (RI) was used as the test AMF inoculum, which was provided by the Agricultural Culture Collection of China. AMF inoculation was performed using the layering method (100 g). First, 100 g of high-temperature sterilized RI inoculation is added to the control treatment to ensure that the composition of other microorganisms is consistent, except for the target microorganism. Prior to treatment, each pot was prepared with 15 established *F. rubra* plants. Once plant growth stabilized, nitrogen application commenced. The experiment employed a completely randomized block design with three factors: nitrogen, AMF, and endophytic fungi. Specific treatments included inoculation with *R. irregularis* (RI) versus no inoculation (NM); infection with endophytic fungi (E+) versus no infection (E-); and three nitrogen levels: (HN, 3 g/L), low nitrogen (LN, 0.3 g/L), and no nitrogen (N0, 0 g/L). Urea was used in the experiments, and the nitrogen concentration was maintained during the growing experiment via periodic nitrogen application (supplemented every ten days). Each treatment was replicated three times, totaling 36 pots. Hoagland nutrient solution was periodically applied to sustain healthy plant growth. Each pot received 500 mL of a modified Hoagland solution every two weeks to ensure a consistent nutritional baseline across treatments. The Hoagland solution contained 5.0 mM CaCl_2_, 5.0 mM KCl, 2.5 mM MgSO_4_·7H_2_O, 2.0 mM KH_2_PO_4_, 29 μM Na_2_-EDTA, 20 mM FeSO_4_·7H_2_O and trace elements, 45 mM H_3_BO_3_, 6.6 mM MnSO_4_, 0.8 mM ZnSO_4_·7H_2_O, 0.6 mM H_2_MoO_4_, and 0.4 mM CuSO_4_·5H_2_O. After a 90-day cultivation period, mycorrhizal colonization rate, plant growth, and physiological parameters were measured. The experimental design diagram is illustrated in Figure 5.

### 4.2. Research Methods

#### 4.2.1. Measurement of Plant Photosynthetic Parameters and Nutrient Characteristics

Measurements were conducted on a clear, windless morning between 9:00 and 11:00 a.m. to assess plant intercellular CO_2_ concentration (Ci), stomatal conductance (Gs), vapor pressure deficit (VPD), net photosynthetic rate (A), transpiration rate (Tr), and water use efficiency (WUE) using a photosynthesis analyzer (CIRAS-3). Total chlorophyll content, chlorophyll a, chlorophyll b, and carotenoid content in plant leaves were extracted using the acetone extraction method. Plant samples were oven-dried and ground using a ball mill. An elemental analyzer (vario MACRO cube from Elementar, Hanau, Germany) was used to determine total carbon (STC), total nitrogen (STN), and total sulfur (STS) in the aboveground parts of *F. rubra*, as well as root total carbon (RTC), total nitrogen (RTN), and total sulfur (RTS).

#### 4.2.2. Determination of Enzyme Activity in Rhizosphere Soil

The shaking method was employed to collect rhizosphere soil. Soil adhering to the root system was shaken off and collected as rhizosphere soil. *F. rubra* plants and soil were removed from pots. The soil attached to the roots was shaken off and collected into a self-sealing bag, ensuring thorough mixing to obtain rhizosphere soil samples. After collection, the plant roots were gently rinsed with sterile water. The roots were then cut at the stem–root junction, separating the above-ground parts (stems, leaves, etc.) from the underground parts (roots), which were placed into pre-weighed envelopes. The fresh weight of each part was recorded before drying for storage. Soil enzyme activity was measured using the ELISA method for four enzymes involved in carbon, nitrogen, and phosphorus cycles: N-acetyl-β-D-glucosidase (S-NAG), β-glucosidase (β-GC), leucine aminopeptidase (L-LAP), neutral phosphatase (NP), and two oxidases, polyphenol oxidase (PPO) and peroxidase (POD). Enzyme activity measurements were conducted at Shengong Biotechnology Co., Ltd., Shanghai, China.

#### 4.2.3. Metagenomic Sequencing and Analysis of Rhizosphere Soil Microbial Communities

Sample DNA extraction was performed using the E.Z.N.A.^®^ Soil DNA Kit (Omega Bio-tek, Norcross, GA, USA). DNA was sheared using the Covaris M220 (GeneTech, Shanghai, China), and fragments of approximately 400 bp were selected for paired-end (PE) library construction. FastP [7] (version 0.20.0) was used to remove reads shorter than 50 bp, with an average base quality score below 20, or containing N bases, retaining only high-quality fragments. Contaminated fragments with high similarity to the host were removed using BWA [58] (version 0.7.9a). Optimized sequences were assembled using MEGAHIT [59] (version 1.1.2). The assembly results were predicted using Prodigal [60] and MetaGene [61]. Genes with nucleotide lengths ≥ 100 bp were selected and translated into amino acid sequences. Diamond [62] (version 0.8.35) aligned the amino acid sequences from the non-redundant gene set with the non-redundant protein sequences (NR) database to derive species annotations. Subsequently, species abundance was calculated by summing the gene abundances associated with each species.

### 4.3. Sequence Quality Control and Genome Assembly

The data were analyzed on a free online platform, the Majorbio Cloud Platform. Briefly, paired-end Illumina reads were trimmed of adaptors, and low-quality reads (length < 50 bp or with a quality value < 20 or having N bases) were removed using fastp (version 0.20.0. Metagenomic data were assembled using MEGAHIT (https://github.com/voutcn/megahit, version 1.1.2 (accessed on 10 December 2024)). Contigs with a length ≥ 300 bp were selected as the final assembly result, and the contigs were subsequently used for further gene prediction and annotation.

Statistical data analysis was performed using SPSS 22.0. Experimental results were analyzed with multi-way ANOVA and one-way ANOVA, with Duncan’s multiple-range test for post hoc comparisons. Soil microbial community diversity was calculated using Mothur (1.48.3). Statistical results were visualized using Origin 2021 and Canoco 4.5.

## 5. Conclusions

The complex interactions among AMF, endophytic fungi, and nitrogen availability emerged as critical factors influencing underground total carbon content, transpiration rates, intercellular carbon dioxide concentrations, and the activity of soil extracellular enzymes in *F. rubra*. AMF enhanced the richness of rhizosphere bacterial communities under low-nitrogen conditions, while endophytic fungal infections increased bacterial diversity. Future studies should further investigate the transcriptomic characteristics of rhizosphere microbial communities influenced by AMF and endophytic fungi. This study significantly advances our understanding of the synergistic mechanisms governing the interactions among AMF, endophytic fungi, and their host plants.

## Figures and Tables

**Table 1:** Multifactor variance analyses of the α diversity levels of bacteria, fungi, and archaea in the rhizosphere soil of *F. rubra*.

Treatments	Bacteria						Fungi						Archaea					
	Sobs		Shannon		Simpson		Sobs		Shannon		Simpson		Sobs		**Shannon**		**Simpson**	
	F	P	F	P	F	P	F	P	F	P	F	P	F	P	F	P	F	P
N	0.001	0.999	0.217	0.806	0.276	0.762	0.055	0.946	0.847	0.441	0.775	0.472	0.063	0.939	5.477	0.011	4.049	0.031
AMF	1.123	0.300	0.864	0.362	1.084	0.308	0.293	0.593	6.163	0.020	4.166	0.052	2.155	0.155	9.253	0.006	0.011	0.918
E	0.071	0.793	2.173	0.153	3.172	0.088	0.664	0.423	0.100	0.755	0.077	0.783	0.135	0.717	1.472	0.237	0.226	0.638
N × AMF	2.767	0.083	2.281	0.124	2.086	0.146	2.113	0.143	9.009	0.001	7.372	0.003	3.556	0.044	2.085	0.146	0.467	0.632
N × E	3.004	0.069	4.160	0.028	1.725	0.200	0.305	0.740	2.424	0.110	2.231	0.129	4.061	0.030	0.523	0.599	0.094	0.911
AMF × E	1.160	0.292	0.486	0.493	0.197	0.661	0.481	0.495	0.820	0.374	1.504	0.232	0.094	0.762	1.329	0.260	0.848	0.366
N × AMF × E	0.907	0.417	1.393	0.268	2.079	0.147	0.421	0.661	4.045	0.031	3.201	0.059	0.680	0.516	1.802	0.187	0.038	0.962

**Table 2:** One-way ANOVA of the α diversity of bacteria, fungi, and archaea in rhizosphere soil infected with endophytic fungi and AMF under different treatments. Different lowercase letters indicate significant differences (*p* < 0.05).

Treatments	Bacteria			Fungi			Archaea		
	Sobs	Shannon	Simpson	Sobs	Shannon	Simpson	Sobs	Shannon	Simpson
LN_RI/E−	3214.330 ± 49.217 ab	5.090 ± 0.307 bc	0.029 ± 0.014 a	290.000 ± 52.048 a	2.505 ± 1.039 bc	0.327 ± 0.25 abcd	209.000 ± 11.136 bc	3.059 ± 0.107 bcd	0.102 ± 0.016 a
LN_NM/E+	3219.670 ± 28.537 ab	5.495 ± 0.030 a	0.011 ± 0.001 b	305.330 ± 6.351 a	2.243 ± 0.222 c	0.389 ± 0.037 ab	214.670 ± 5.508 abc	3.318 ± 0.132 a	0.098 ± 0.013 a
LN_RI/E+	3241.670 ± 22.143 ab	5.407 ± 0.027 abc	0.014 ± 0.002 ab	282.330 ± 15.308 a	3.741 ± 0.649 ab	0.110 ± 0.098 bcd	218.000 ± 2.000 ab	3.003 ± 0.088 bcd	0.099 ± 0.011 a
LN_NM/E−	3213.330 ± 34.429 ab	5.357 ± 0.141 abc	0.014 ± 0.002 ab	301.000 ± 57.166 a	2.399 ± 1.377 bc	0.360 ± 0.301 abc	210.000 ± 8.544 abc	3.247 ± 0.121 ab	0.094 ± 0.013 a
N0_RI/E−	3250.330 ± 16.503 a	5.343 ± 0.097 abc	0.016 ± 0.003 ab	297.000 ± 11.000 a	2.871 ± 0.477 abc	0.265 ± 0.083 bcd	214.670 ± 2.517 abc	2.953 ± 0.127 cd	0.107 ± 0.018 a
N0_NM/E+	3219.670 ± 31.66 ab	5.432 ± 0.054 ab	0.013 ± 0.001 ab	280.670 ± 27.610 a	3.985 ± 0.554 a	0.080 ± 0.079 cd	215.330 ± 7.572 ab	3.049 ± 0.049 bcd	0.111 ± 0.012 a
N0_RI/E+	3246.330 ± 11.015 ab	5.393 ± 0.075 abc	0.016 ± 0.003 ab	310.000 ± 20.664 a	2.477 ± 1.117 bc	0.357 ± 0.249 abcd	212.670 ± 2.517 abc	3.113 ± 0.041 abc	0.095 ± 0.003 a
N0_NM/E−	3174.670 ± 98.5 b	5.043 ± 0.506 c	0.028 ± 0.023 a	275.330 ± 68.237 a	2.86 ± 0.789 abc	0.224 ± 0.158 bcd	211.330 ± 6.028 abc	3.134 ± 0.036 abc	0.109 ± 0.001 a
HN_RI/E−	3248.330 ± 3.786 ab	5.397 ± 0.088 abc	0.016 ± 0.004 ab	278.330 ± 11.719 a	3.924 ± 0.363 a	0.077 ± 0.045 cd	211.330 ± 1.528 abc	2.847 ± 0.209 d	0.127 ± 0.031 a
HN_NM/E+	3223.000 ± 17.321 ab	5.362 ± 0.164 abc	0.014 ± 0.005 ab	324.000 ± 7.81 a	1.487 ± 0.391 c	0.559 ± 0.079 a	215.000 ± 1.732 abc	3.035 ± 0.127 bcd	0.120 ± 0.015 a
HN_RI/E+	3173.670 ± 20.429 b	5.179 ± 0.105 abc	0.020 ± 0.004 ab	277.000 ± 8.888 a	3.974 ± 0.182 a	0.056 ± 0.019 d	204.670 ± 3.215 c	3.045 ± 0.243 bcd	0.116 ± 0.045 a
HN_NM/E−	3243.330 ± 28.095 ab	5.475 ± 0.021 a	0.014 ± 0.002 ab	284.000 ± 22.539 a	2.887 ± 0.493 abc	0.211 ± 0.121 bcd	220.000 ± 1.732 a	3.012 ± 0.058 bcd	0.117 ± 0.003 a

## Data Availability

The raw data supporting the conclusions of this article will be made available by the authors upon request.
